# Severity of respiratory syncytial virus through the COVID-19 pandemic among infants aged ≤2 months: a secondary analysis of the IRIDE cohort study

**DOI:** 10.1038/s41390-025-04161-3

**Published:** 2025-06-10

**Authors:** Andrea Ronchi, Gregorio Paolo Milani, Carlo Agostoni, Paola Marchisio, Giovanna Chidini, Nicola Pesenti, Riccardo Crimi, Elena Gariboldi, Anita Bellotti, Marco Cugliari, Valentina Fabiano, Carlo Pietrasanta, Fabio Mosca, Lorenza Pugni, Monica Fumagalli

**Affiliations:** 1 Neonatal Intensive Care Unit, Fondazione IRCCS Ca’ Granda Ospedale Maggiore Policlinico, Milan, Italy; 2 Pediatric Unit, Fondazione IRCCS Ca’ Granda Ospedale Maggiore Policlinico, Milan, Italy; 3 Department of Clinical Sciences and Community Health, Dipartimento di Eccellenza 2023-2027, Università degli Studi di Milano, Milan, Italy; 4 Department of Pathophysiology and Transplantation, University of Milan, Milan, Italy; 5 Department of Anaesthesia, Intensive Care and Emergency, Fondazione IRCCS Ca’ Granda Ospedale Maggiore Policlinico, Milan, Italy; 6 Università Degli Studi Di Milano, Milan, Italy; 7 Pediatric Department, “Vittore Buzzi” Children’s Hospital, Milan, Italy; 8 Department of Biomedical and Clinical Science, University of Milan, Milan, Italy

## Abstract

**Background:**

Through the SARS-CoV-2 pandemic the severity of RSV infection increased among children, but little is known about its evolution among infants ≤60 days of life (DOL).

**Methods:**

Multicenter observational retrospective study conducted in 27 hospitals in Lombardy, Italy, comparing outcomes of infants (≤60 DOL) hospitalized with RSV bronchiolitis during 4 seasons: 2018-2019 (pre-pandemic period), 2020–2023 (pandemic seasons).

**Result:**

1816 infants were included. Multivariable regressions showed that the need and length of O_2_-supplementation were significantly higher in pandemic seasons compared to the pre-pandemic one, as the need for non-invasive ventilation. Among neonates (≤28 DOL), no difference was observed between pre- and pandemic seasons besides an increased length of O_2_-supplementation.

**Conclusion:**

Outcomes of RSV bronchiolitis among infants ≤60 DOL were slightly affected by COVID-19 pandemic. The impact was less significant among neonates. Breast milk components might have mitigated the negative effect of COVID-19 on RSV severity in neonates.

**Category of study:**

Clinical and population study.

**Impact:**

- Outcomes of RSV bronchiolitis among infants ≤60 days of life (DOL) were slightly affected by COVID-19 pandemic. Neonates (≤28 DOL) in our cohort seem to have significantly better outcomes than older infants (29-60 DOL).
- To date no data is available on the effects of non-pharmacological interventions on the diffusivity and severity of RSV infection in infants aged ≤60 DOL, the population at highest risk for RSV-related complications
- Breast milk and its innate immune components may have played an important role as protective factors against RSV respiratory infections during the first month of life

## Introduction

Respiratory syncytial virus (RSV) is the most frequent cause of lower respiratory tract infections (LRTI) in children, and the leading cause of hospitalization in the first months of life worldwide.^1–3^ RSV causes approximately 33 million of LRTI per year in infants younger than 5 years of age, 10% of whom require hospitalization;^1–4^ among them, infants in the first 2 months of life have the highest RSV-related hospitalization rate, ranging from 17.7 to 31.2 per 1000 infants.^2–4^

In March 2020 the rapid propagation of the SARS-CoV-2 pandemic led most health authorities worldwide to enforce non-pharmacological interventions (NPIs) such as “lock-down”, universal face covering, and social distancing to control the spread of the virus. Despite effective in reducing the impact of SARS-CoV-2 on global health^5,6^ the implementation of NPIs was followed by unpredicted off-season severe epidemic outbreaks of several respiratory viruses, including RSV.^6–8^ The reason of this resurgence has been frequently ascribed to the concept of “immune debt” or “immune shortage”,^9^ a term that describes the reduction of antigen-specific immune response in people not exposed to pathogens during the period of NPIs implementation.

Published scientific literature to date highlighted that NPIs have favored off-season severe epidemic outbreaks of RSV, and a shift in RSV-related severity to older children (24-59 months) during the seasons following the acute phase of SARS-CoV-2 pandemic (year 2020) compared to previous years.^5–7,10^ Most research has been conducted in heterogeneous populations of children aged <5 years, while no data is available on the effects of NPIs on the diffusivity and severity of RSV infection in infants aged ≤60 days of life (DOL), the population at highest risk for RSV-related complications.

Thus, our study aimed to investigate the effect of NPIs implemented in 2020 to limit the circulation of SARS-CoV-2 on the diffusivity and severity of RSV infection in infants ≤60 DOL, a narrow and homogeneous population at the highest risk of RSV-related hospitalization. As secondary analysis, we investigated the outcomes of RSV infection before and after the acute phase of pandemic among neonates (≤28 DOL) and young infants (29 to 60 DOL).

## Methods

This is a secondary analysis of the Italian IRIDE (“Investigating bRonchIolitis epidemiology During the pandemic Emergency”) study, a multicenter observational retrospective cohort of children recruited in 27 hospitals in Lombardy, Italy with an age less than 24 months and hospitalized for at least 12 h with a diagnosis of bronchiolitis.^8,11^

Infants younger than 24 months, hospitalized from July to March: 2018–2019 (pre-pandemic period), 2020- 2021, 2021–2022, and 2022- 2023 with a diagnosis of bronchiolitis, caused by any viruses, were included in the main study. The study protocol was approved by the Ethical Committee of the coordinating center (Comitato Etico Milano Area2, Fondazione IRCCS Ca’ Granda Ospedale Maggiore Policlinico, Milano, code 186796, date of approval April 26, 2023) and of the other participating centers. All study procedures were conducted in accordance with the Declaration of Helsinki.^12^

Since the outbreak of SARS-CoV-2 and the first restrictive measures to limit its spread were applied in Lombardy at the beginning of 2020, the period 2019–2020 was not considered. The three pandemic periods considered in this study were characterized by a gradual decrease in preventive and hygiene measures against viral circulation.^13,14^

The current NeoIRIDE study recruited all hospitalized infants younger than 60 DOL with RSV bronchiolitis, while infants with bronchiolitis caused by non-RSV viruses and those older than 60 DOL were excluded.

Eligible patients were identified by the following International Classification of Disease, 9^th^ edition (ICD-9) codes at hospital discharge: 46.611 (respiratory syncytial virus bronchiolitis), 4801 (pneumonia due to respiratory syncytial virus), 0796 (respiratory syncytial virus infection), 51.881 (respiratory failure in children), 77.084 and 77.089 (respiratory failure in neonates).

All medical records were reviewed, and pertinent demographic and clinical data were recorded.

The following data were retrospectively extracted from the clinical records: age at admission, sex, neonatal gestational age (term neonates were defined as those born at a gestational age (GA) between 37 and 42 weeks; preterm neonates were defined as those born at a GA less than 37 weeks), birth weight, any breastfeeding, number of older siblings, Palivizumab prophylaxis, need and duration of oxygen supplementation, use of non-invasive and invasive ventilation support, use of extracorporeal membrane oxygenation (ECMO), need and duration of intensive care, length of the whole hospital stay. Furthermore, fatal cases were also considered. All data were gathered and de-identified at each study center. They were then collected using the REDCap web-based platform hosted at the Fondazione IRCCS Ca’ Granda Ospedale Maggiore, Policlinico, Milan, Italy.

## Statistical analysis

The distribution of continuous variables were presented as mean and standard deviation (SD) when normally distributed or as median and interquartile range [IQR] when their distribution was non-normal. Categorical data were presented as absolute and relative frequencies. Differences in continuous variables between groups were investigated by *t* test or Mann–Whitney U test, while one-way ANOVA for repeated measures and Friedman’s test were used for comparison between the three periods. Fisher’s exact test or Chi-square test were used for categorical variables. Mixed-effects models were used to investigate differences in outcomes between the four study periods (2018–19, 2020–21, 2021–22 and 2022–23). Age on admission, gestational age at birth, sex, prematurity, number of siblings and breastfeeding were considered as potential confounders. Model results were expressed as linear coefficients or odds ratios for continuous and categorical outcomes, respectively, with 95% CIs. The categorical outcomes included: admission to intensive care unit, need for invasive ventilation, need for non-invasive ventilation, need for oxygen therapy during admission; the continuous outcomes comprised: duration of oxygen supplementation, stay in intensive care unit, overall duration of hospitalization. A secondary analysis was also performed replicating the models used on the overall population, focusing only on patients aged ≤28 DOL (“neonates”) and between 29 and 60 DOL (“young infants”). Statistical significance was set at p<0.05. Data analyses were performed using R software, version 4.0.1 or higher (R Foundation for Statistical Computing, Vienna, Austria).^15^

## Results

A total of 2572 infants up to 60 DOL with bronchiolitis were hospitalized, 756 of whom were excluded from the analysis since the isolated viruses were other than RSV. Therefore, during the study period (July to March, seasons: 2018–2019, 2020–2021, 2021–2022, 2022–2023) 1816 infants up to 60 DOL were included. No infant with RSV infection was hospitalized in the 2020–2021 season. Overall, 54.4% were male with a median (IQR) age at admission of 30 (25-60) DOL. Most hospitalized infants were born at full term (90.2%) with a mean GA (±SD) of 38.7 (±1.7) weeks and a mean birth weight of 3213 (±505) g. Only 139 (7.6%) were born prematurely (one ≤29 weeks of GA; 80 between 30-35 weeks of GA and 58 between 36 and 37 weeks of GA).

Admitted infants with known risk factors for severe RSV infection, who received Palivizumab prophylaxis, were negligible (18 infants: 9 with congenital heart disease; 6 with Down syndrome; 1 with bronchopulmonary dysplasia; 1 with cystic fibrosis; 1 with neuromuscular disease). Most patients were breastfed (79.8%) and 865 (47.6%) patients had at least one sibling. During the hospitalization, 1476 (81.3%) infants required oxygen supplementation for a median duration of 5 (IQR 3–8) days, 1107 (61%) received non-invasive ventilatory support, 50 (2.8%) required invasive ventilation and only one patient required ECMO. The median duration of hospitalization was 7 (IQR 5–9) days and approximately one quarter of patients (*n *= 484, 26.7%) required admission to the intensive care unit. Two patients died.

Across seasons the number of infants (≤60 DOL) hospitalized increased from 465 in 2018–2019 to 680 in 2022–2023 season even though no significant differences between demographic characteristics of infants were noted (Table 1). Compared to 2018–2019 period, the univariate analysis showed worsening outcomes through the COVID-19 pandemic among infants ≤60 DOL, such as an increase in infants requiring oxygen supplementation (2018–2019: 346 (74.4%); 2021–2022: 562 (83.8%), *p *< 0.001; 2022–2023: 568 (83.5%), *p *< 0.001), and non-invasive ventilation (2018–2019: 231 (49.7%); 2021–2022: 417 (62.1%), *p* < 0.001; 2022–2023: 459 (67.5%), *p *< 0.001) (Supplementary Table S1).

**Table 1:** Demographics and clinical characteristics of infants (≤60 days of life) in the three study periods.

Characteristics	2018–2019	2021–2022	2022–2023	*p*
N	465	671	680	
Age, days [IQR]	30 [29.1, 60]	30 [24.6, 60]	30 [24.9, 60]	0.090
Sex				
Female, n (%)	213 (45.8)	327 (48.7)	288 (42.4)	0.062
Male, n (%)	252 (54.2)	344 (51.3)	392 (57.6)	
Gestational age, weeks (SD)	38.58 (1.93)	38.67 (1.69)	38.67 (1.64)	0.684
Weight, grams (SD)	3183.32 (538.84)	3247.04 (502.73)	3198.68 (482.04)	0.117
Preterm birth (<37 wks), n (%)	44 (12.0)	51 (9.3)	44 (8.8)	0.276
Number of siblings, n [IQR]	1 [0.00, 1.00]	1 [1.00, 1.00]	1 [1.00, 1.00]	0.13
Breastfeeding, n (%)	314 (80.1)	480 (78.0)	443 (81.4)	0.352
Radiologically confirmed pneumonia				
Yes, n (%)	94 (20.2)	114 (17.0)	86 (13.0)	**<0.001**
No, n (%)	149 (32.0)	216 (32.2)	154 (23.2)	
Rx not performed, n (%)	222 (47.7)	341 (50.8)	424 (63.9)	
Antibiotic therapy, n (%)	215 (46.2)	228 (34.0)	217 (31.9)	**<0.001**
Corticosteroid therapy, n (%)	118 (25.4)	197 (29.4)	203 (29.9)	0.221
Discharge ward				
Emergency unit—n (%)	12 (2.6)	10 (1.5)	11 (1.6)	0.075
Neonatal unit—n (%)	106 (22.8)	172 (25.6)	201 (29.6)	
Pediatric unit—n (%)	347 (74.6)	489 (72.9)	468 (68.8)	
Death—n (%)	1 (0.2)	1 (0.1)	0 (0.0)	0.534

Significant *p*-values highlighted in bold.

These results were partly confirmed by the multivariate analysis. Mixed models showed that infants required more oxygen supplementation [2021–2022 (OR, 2.16; 95% CI, 1.3–3.5; *p* = 0.002) and 2022–2023 (OR, 2.03; 95% CI, 1.16–3.55; *p* = 0.013)] and its duration was longer [2021–2022 (coefficient, 1.07; 95% CI, 0.5–1.63; *p* < 0.001) and 2022–2023 (coefficient, 1.04; 95% CI, 0.4–1.68; *p* = 0.001)] in both seasons compared to 2018–2019. Furthermore, more infants required non-invasive ventilation in 2021–2022 (OR, 2.55; 95% CI, 1.7–3.9; *p* < 0.001) and in 2022–2023 (OR, 3.7; 95% CI, 2.26–6; *p* < 0.001), compared to 2018–2019 (Table 2).

**Table 2:** Outcomes of infants (≤60 days of life): results of the mixed effect regression models.

Characteristics	Period	Odds ratio/ mean difference	IC 95%	*p*
Oxygen supplementation	2021–2022	2.157	1.330–3.497	**0.002**
	2022–2023	2.030	1.162–3.549	**0.013**
Length of oxygen supplementation	2021–2022	1.068	0.503–1.633	**<0.001**
	2022–2023	1.042	0.403–1.680	**0.001**
Non invasive ventilation	2021–2022	2.550	1.665–3.906	**<0.001**
	2022–2023	3.700	2.262–6.053	**<0.001**
Invasive ventilation	2021–2022	0.407	0.123 - 1.351	0.142
	2022–2023	1.656	0.624–4.392	0.311
Length of hospital stay	2021–2022	−0.255	–0.866 to 0.356	0.413
	2022–2023	0.231	–0.459 to 0.921	0.511
Admission to the intensive care unit	2021–2022	1.329	0.801–2.206	0.271
	2022–2023	1.344	0.740–2.440	0.331
Length of stay in the intensive care unit	2021–2022	0.032	–0.451 to 0.515	0.897
	2022–2023	0.162	–0.389 to 0.712	0.564

Study periods (reference 2018–19) were the predictive variables.

Significant *p*-values highlighted in bold.

Then, we compared the demographic data and outcomes across the study seasons among the sub-populations of neonates (≤28 DOL) and young infants (between 29 and 60 DOL).

In both sub-populations, the number of hospitalizations increased in 2021–2022 and 2022–2023 seasons (Table 3).

**Table 3:** Demographics and clinical characteristics of neonates (≤28 days of life) and young infants (29–60 days of life) in the three study periods.

Characteristics	2018–2019		2021–2022		2022–2023		p^1^	p^2^
Neonates/Young infants	≤28 DOL	29–60 DOL	≤28 DOL	29–60 DOL	≤28 DOL	29–60 DOL		
*N*	111	354	209	462	211	469		
Age, days [IQR]	18 [14.1, 23.1]	45 [30, 60]	18.9 [15, 24]	45 [30, 60]	18 [12.9, 23.1]	45 [30, 60]	0.192	0.387
Sex								
Female, n (%)	60 (54.1)	153 (43.2)	106 (50.7)	221 (47.8)	77 (36.5)	211 (45.0)	**0.002**	0.405
Male, n (%)	51 (45.9)	201 (56.8)	103 (49.3)	241 (52.2)	134 (63.5)	258 (55.0)		
Gestational age, weeks (SD)	38.80 (1.57)	38.52 (2.01)	38.95 (1.48)	38.54 (1.77)	38.76 (1.44)	38.62 (1.73)	0.484	0.747
Weight, grams (SD)	3258.53 (495.11)	3159.98 (550.43)	3345.03 (434.56)	3199.19 (526.79)	3239.70 (442.47)	3178.66 (499.63)	0.073	0.628
Preterm birth (<37 wks), n (%)	6 (7.4)	38 (13.2)	13 (7.2)	38 (10.4)	10 (6.1)	34 (10.1)	0.901	0.402
Number of siblings, n [IQR]	1 [0.25, 1.00]	1 [0.00, 1.00]	1 [1.00, 2.00]	1 [1.00, 1.00]	1 [1.00, 1.00]	1 [0.00, 1.00]	0.069	0.093
Breastfeeding, n (%)	74 (77.9)	240 (80.8)	170 (84.6)	310 (74.9)	140 (80.5)	303 (81.9)	0.331	**0.036**
Radiologically confirmed pneumonia								
Yes, n (%)	26 (23.4)	68 (19.2)	40 (19.1)	74 (16.0)	28 (13.3)	58 (12.8)	**0.036**	**<0.001**
No, n (%)	32 (28.8)	117 (33.1)	76 (36.4)	140 (30.3)	62 (29.4)	92 (20.3)		
Rx not performed, n (%)	53 (47.7)	169 (47.7)	93 (44.5)	248 (53.7)	121 (57.3)	303 (66.9)		
Antibiotic therapy, n (%)	55 (49.5)	160 (45.2)	69 (33.0)	159 (34.5)	73 (34.6)	144 (30.7)	**0.009**	**<0.001**
Corticosteroid therapy, n (%)	21 (18.9)	97 (27.5)	32 (15.3)	165 (35.8)	37 (17.5)	166 (35.4)	0.685	**0.022**
Discharge ward								
Emergency unit - n (%)	1 (0.9)	11 (3.1)	1 (0.5)	9 (1.9)	0 (0.0)	11 (2.3)	0.568	0.176
Neonatal care unit - n (%)	56 (50.5)	50 (14.1)	117 (56.0)	55 (11.9)	121 (57.3)	80 (17.1)		
Pediatric - n (%)	54 (48.6)	293 (82.8)	91 (43.5)	398 (86.1)	90 (42.7)	378 (80.6)		
Death - n (%)	0	1 (0.3)	0	1 (0.2)	0	0 (0.0)	0	0.56

p1: comparison of neonates (≤28 DOL) characteristics.

p2: comparison of young infants (29–60 DOL) characteristics.

Significant *p*-values highlighted in bold.

In the neonatal population, (≤28 DOL) the univariate analysis did not show worse outcomes in the 2021–2022 and 2022–2023 seasons compared to the 2018–2019 season (Supplementary Table S2) while the multivariate analysis showed that only the duration of oxygen supplementation was longer in both 2021–2022 (coefficient 1.89; 95% CI, 0.67–3.12; *p* = 0.002) and 2022–2023 (coefficient 2; 95% CI, 0.69–3.3; *p* = 0.003) seasons (Table 4).

**Table 4:** Outcomes of neonates (≤28 days of life): results of the mixed effect regression models.

Characteristics	Period	Odds ratio/ mean difference	IC 95%	*p*
Oxygen supplementation	2021–2022	1.125	0.320–3.958	0.854
	2022–2023	1.121	0.292–4.306	0.867
Length of oxygen supplementation	2021–2022	1.898	0.676–3.120	**0.002**
	2022–2023	2.001	0.697–3.305	**0.003**
Non invasive ventilation	2021–2022	1.684	0.626–4.531	0.302
	2022–2023	2.193	0.760–6.331	0.147
Invasive ventilation	2021–2022	0.207	0.017–2.458	0.212
	2022–2023	1.979	0.360–10.89	0.433
Length of hospital stay	2021–2022	−0.185	−1.481 to 1.111	0.778
	2022–2023	−0.126	−1.512 to 1.259	0.858
Admission to the intensive care unit	2021–2022	0.752	0.277–2.040	0.575
	2022–2023	0.711	0.243–2.086	0.535
Length of stay in the intensive care unit	2021–2022	−0.221	−1.459 to 1.016	0.725
	2022–2023	–0.304	–1.632 to 1.025	0.653

Study periods (reference 2018–19) were the predictive variables.

Significant *p*-values highlighted in bold.

Conversely, in the population of young infants aged 29–60 DOL the univariate analysis showed significantly worsening outcomes in 2021–2022 and 2022–2023 seasons compared to 2018–2019: indeed, young infants required more oxygen supplementation (2018–2019: 247 (69.8%); 2021–2022: 376 (81.4%), *p* <  0.001; 2022–2023: 384 (81.9%), *p* < 0.001) and more non-invasive ventilation (2018–2019: 155 (43.8%); 2021–2022: 253 (54.8%), *p* = 0.002; 2022–2023: 296 (63.1%), *p* < 0.001) (Supplementary Table S3). The multivariate analysis confirmed an increased need for oxygen supplementation in both 2021–2022 (OR, 2.52; 95% CI, 1.45–4.38; *p* = 0.001) and 2022–2023 (OR, 2.16; 95% CI, 1.13–4.15; *p *= 0.02) seasons and the overall duration of O_2_ supplementation was longer in the 2021–2022 (coefficient, 0.86; 95% CI, 0.24–1.49; *p *= 0.007) season. Finally, young infants admitted for RSV bronchiolitis had an increased risk of requiring non-invasive ventilation in both 2021–2022 (OR, 2.89; 95% CI, 1.76–4.75; *p *< 0.001) and 2022–2023 (OR, 4.15; 95% CI, 2.33–7.39; *p *< 0.001) seasons (Table 5) compared to the pre-pandemic period.

**Table 5:** Outcomes of young infants (29–60 days of life): results of the mixed effect regression models.

Characteristics	Period	Odds ratio/ mean difference	IC 95%	*p*
Oxygen supplementation	2021–2022	2.515	1.445–4.378	**0.001**
	2022–2023	2.164	1.129–4.149	**0.02**
Length of oxygen supplementation	2021–2022	0.864	0.237–1.491	**0.007**
	2022–2023	0.679	–0.054 to 1.412	0.069
Non invasive ventilation	2021–2022	2.891	1.760–4.748	**<0.001**
	2022–2023	4.152	2.334–7.386	**<0.001**
Invasive ventilation	2021–2022	0.605	0.153–2.388	0.473
	2022–2023	1.703	0.520–5.576	0.379
Length of hospital stay	2021–2022	-0.331	–1.022 to 0.359	0.347
	2022–2023	0.24	–0.565 to 1.045	0.558
Admission to the intensive care unit	2021–2022	1.519	0.804–2.870	0.198
	2022–2023	1.701	0.781 to 3.702	0.181
Length of stay in the intensive care unit	2021–2022	0.044	–0.436 to 0.524	0.857
	2022–2023	0.232	–0.335 to 0.798	0.422

Study periods (reference 2018–19) were the predictive variables.

Significant *p*-values highlighted in bold.

## Discussion

The NeoIRIDE study showed that the introduction of non-pharmacological interventions (“lock-down”, face covering, and social distancing) introduced during the SARS-CoV-2 pandemic was followed by an increase in the hospital admissions due to RSV infection in infants ≤60 DOL during the two seasons 2021–2022 and 2022–2023 versus the pre-pandemic 2018–2019 season.

A higher number of RSV-associated hospital admissions in children during the epidemic seasons 2021–2022 and 2022–2023 compared to pre-COVID seasons has been previously reported in several other countries.^6,16–20^ This phenomenon has been ascribed to the so-called RSV-specific “immune debt”, a comprehensive term describing the diminished antigen-specific immune response caused by limited exposure to the virus during the extended period of NPIs application (2020; lock-down period).^21–23^

Concerning the severity of the infection, we observed slightly worse outcomes in RSV-infected infants (≤60 DOL) after the acute phase of COVID-19 pandemic as compared to the pre-pandemic seasons. Multivariate analysis demonstrated that the infant population (≤60 DOL) just needed more oxygen supplementation for a longer time, as well as more non-invasive ventilation. These findings have already been partly reported by other authors in more heterogeneous populations of older children. Garcia-Maurino et al. observed that children aged <1 year required more oxygen supplementation in the pandemic period compared to the pre-pandemic one, and similar results were reported by Milani et al. who observed a higher odds for oxygen requirement in children aged <2 years during the pandemic seasons. Ghirardo et al. also observed a statistically significant increase of oxygen supplementation in children aged <1 year in the 2021–2022 season as compared with the pre-pandemic season.^8,10,24^ In our population of RSV-infected infants, the length of oxygen therapy was also affected by the COVID-19 pandemic. Indeed, infants ≤60 DOL needed on average one more day of oxygen therapy in the 2021–2022 and 2022–2023 seasons compared with the 2018–2019 season. Likewise, Milani et al. reported similar data.^8^ Finally, Ghirardo et al. and Milani et al. also observed a significantly increased need for non-invasive respiratory support in RSV-infected children during the pandemic seasons compared to the pre-pandemic, as we noticed in infants ≤60 DOL.

Despite the aforementioned similarities, our study differed from most previously published literature, as we sought to focus exclusively on a narrower and more homogeneous population of infants, that at higher risk for hospitalization due to RSV infection.^1,2,25^ Indeed, most previously analyzed cohorts of RSV-infected children encompassed broader age ranges, up to 5 years of age, clearly showing that older children in these groups, between 2 and 5 years of age, experienced the most significant worsening of outcomes (i.e. admission to intensive care unit, need for mechanical ventilation) after the acute phase of COVID-19 pandemic. This possibly occurred because of postponed primary RSV infection in older children, that exposed them to previously unexperienced severe consequences. Interestingly, we have not observed such a significant increase of very severe cases of RSV bronchiolitis in our cohort of infants. The different composition of our cohort might justify the discrepancies between our relatively milder worsening of outcomes after the acute phase of COVID-19 pandemic and those reported by other authors.^5–7,10,24^ Indeed, apart from the protective role of maternal antibodies or passive immunization, infants do not routinely benefit from pre-existing antigen-specific immunity against RSV: thus, the negative effects of NPIs on RSV-specific immunity might have been milder in our population.

Moreover, evaluating the impact of COVID-19 pandemic and NPIs on RSV-related outcomes in the two sub-populations of neonates (≤28 DOL) and young infants (29–60 DOL), we highlighted that neonates were even less affected by the introduction of NPIs. Neonates exclusively required on average two more days of oxygen therapy after the acute phase of the pandemic, data that are consistent with the results described by Akbay Ak et al., the only ones who have described a population of neonates.^26^

Conversely, young infants (29–60 DOL) had an increased need for oxygen supplementation in both seasons following the acute phase of COVID-19 pandemic, an oxygen supplementation that lasted for longer, and a significantly increased risk of requiring non-invasive ventilation. These findings recalled, partially, those observed in more heterogeneous and older populations described in the literature and mentioned before.^8,10,24^

It is known in principle that younger infants are at higher risk for severe RSV-related outcomes as compared with older children, and that chronological age is inversely correlated with the probability of being hospitalized or receiving intensive care due to RSV infection.^1^ However, it seems from our data that neonates ≤28 DOL presented a reduced worsening of RSV-related outcomes after the acute phase of COVID-19 pandemic as compared to older infants, specifically those infected during the 2^nd^ month of life.

Robust evidence from published literature indicates that the main protective factors for reducing the risk of RSV infection early in life are the administration of Palivizumab, in at-higher risk infants, and maternal breastfeeding.^27^ The protective role of Palivizumab was not assessable in our cohort because only 18 infants were eligible to receive it according to local guidelines.^2^ Conversely, most neonates and young infants in our cohort were breastfed at the moment of RSV infection, consistently throughout the three seasons considered. We speculate that maternal breastfeeding may have played an important role in shaping the outcomes of our cohort. The beneficial effects of breastfeeding in reducing the risk of respiratory infections, particularly those caused by RSV is well known,^28,29^ and a large meta-analysis of 20 independent studies showed that infants not breastfed have odds of suffering for RSV-related acute lower respiratory infection 2.24 times greater than breastfed ones (95% CI: 1.56-3.20).^29^

The concentration of antigen-specific anti-RSV immune components of breast milk, such as secretory IgAs (SIgA), remain stable during the first two months of lactation,^30^ not supporting a significant role in shaping the relatively better outcomes of neonates compared to older infants after the acute phase of COVID-19 pandemic.

Together with antigen-specific molecules, non-antigen-specific immune components of breast milk such as cytokines, lactoferrin, and human milk oligosaccharides (HMOs) contribute to the protection of breastfed infants against respiratory viral pathogens.^31–34^ It has been shown that the concentration of most innate immune components of breast milk decreases after 3–4 weeks of lactation.^35–38^ Therefore, we speculate that non-antigen-specific immune components of breast milk might have been at least partially responsible for the absence of worsening of RSV-related outcomes in the neonatal population (≤28 DOL), but not in the infant one (29–60 DOL), after the onset of COVID-19 pandemic.

Our study has some limitations, which include the retrospective design, the inclusion of patients secondary to their identification by ICD-9 codes (whose accuracy can be suboptimal), and the lack of standardized clinical approaches at the different study sites. The study also has strengths: its multicenter nature, the large number of patients enrolled and, above all, being focused on the narrow and at-highest-risk homogeneous population of infants (≤60 DOL). Finally, the stratification of the cohort according to postnatal age in neonates and young infants allowed for the first time a comparison of the outcomes between these two groups.

In conclusion, our study highlighted for the first time that the implementation of COVID-19-related NPIs in Northern Italy in early 2020 was followed by slightly worse outcomes in infants (0-60 DOL) hospitalized with RSV infection in the 2021–2022 and 2022–2023 seasons, compared with the 2018–2019 season. However, this phenomenon was not as severe as that already described in older children. Furthermore, neonates (≤28 DOL) in our cohort seem to have significantly better outcomes than older infants after the acute phase of COVID-19 pandemic. These findings could pave the pathway to more mechanistic research on how the pandemic affected antiviral immune mechanisms in neonates and infants.

## Supplementary information

Supplementary Information

IRIDE_studygroup

## Data Availability

Complete datasets will be available upon reasonable request to the corresponding author
