# Supplementary material for: Severity of respiratory syncytial virus through the COVID-19 pandemic among infants aged ≤2 months: a secondary analysis of the IRIDE cohort study
Source: Pediatr Res. 2025 Jun 10;99(2):663–9. doi: 10.1038/s41390-025-04161-3 (PMC12956566; doi:10.1038/s41390-025-04161-3)
Supplement: Supplementary file 1 — Supplementary Information [file 41390_2025_4161_MOESM1_ESM.pdf]

Supplementary

The following three tables describe the outcomes of children (≤ 60 days of life), neonates (≤ 28 days of life) and young infants (29-60 days of life) in the three seasons considered [2018-2019 (pre-pandemic period), 2021-2022 and 2022-2023 (pandemic seasons)].

Outcomes in 2018-2019 are compared to those in 2021-2022 (p^1) and 2022-2023 (p^2). Data are presented as median [IQR] or absolute frequency (percentage). Differences in continuous variables between groups were investigated by Mann-Whitney U test, and Fisher's exact test or Chi-square test were used for categorical variables.

Supplementary Table S1. Outcomes of infants (≤ 60 days of life) in the three study periods

Characteristics	2018-2019	2021-2022	2022-2023	p^1	p^2
N	465	671	680		
Oxygen supplementation, n (%)	346 (74.4)	562 (83.8)	568 (83.5)	**<0.001**	**<0.001**
Length of oxygen supplementation, days [IQR]	5 [3.25, 8.00]	5 [4.00, 7.00]	5 [3.00, 7.00]	0.497	0.204
Non invasive ventilation, n (%)	231 (49.7)	417 (62.1)	459 (67.5)	**<0.001**	**<0.001**
Invasive ventilation, n (%)	15 (3.2)	9 (1.3)	26 (3.8)	0.05	0.709
Length of hospital stay, days [IQR]	7 [5.00, 9.00]	7 [5.00, 9.00]	7 [5.00, 9.00]	0.312	0.748
Admission to the intensive care unit	117 (25.2)	174 (25.9)	193 (28.4)	0.823	0.256
Length of stay in the intensive care unit, days [IQR]	5 [4.00, 8.00]	5 [3.00, 7.00]	6 [4.00, 8.75]	0.340	0.212

p^1: season 2021-2022 vs. season 2018-2019
p^2: season 2022-2023 vs. season 2018-2019

Supplementary Table S2. Outcomes of neonates (≤28 days of life) in the three study periods

Characteristics	2018-2019	2021-2022	2022-2023	p^1	p^2
N	111	209	211		
Oxygen supplementation, n (%)	99 (89.2)	186 (89.0)	184 (87.2)	0.999	0.734
Length of oxygen supplementation, days [IQR]	5 [4.00, 8.00]	6 [4.00, 8.00]	5 [4.00, 8.00]	0.319	0.675
Non invasive ventilation, n (%)	76 (68.5)	164 (78.5)	163 (77.3)	0.067	0.114

Invasive ventilation, n (%)	4 (3.6)	2 (1.0)	15 (7.1)	0.219	0.308
Length of hospital stay, days [IQR]	8 [6.00, 10.00]	8 [5.00, 10.00]	8 [5.00, 11.00]	0.27	0.526
Admission to the intensive care unit	41 (36.9)	85 (40.7)	92 (43.6)	0.596	0.3
Length of stay in to the intensive care unit, days [IQR]	6 [5.00, 7.00]	5 [4.00, 7.75]	7 [4.75, 9.00]	0.523	0.052

p^1: season 2021-2022 vs. season 2018-2019
p^2: season 2022-2023 vs. season 2018-2019

Supplementary Table S3. Outcomes of young infants (29-60 days of life) in the three study periods

Characteristics	2018-2019	2021-2022	2022-2023	p^1	p^2
N	354	462	469		
Oxygen supplementation, n (%)	247 (69.8)	376 (81.4)	384 (81.9)	**<0.001**	**<0.001**
Length of oxygen supplementation, days [IQR]	5 [3.00, 8.00]	5 [3.00, 7.00]	5 [3.00, 7.00]	0.110	0.150
Non invasive ventilation, n (%)	155 (43.8)	253 (54.8)	296 (63.1)	**0.002**	**<0.001**
Invasive ventilation, n (%)	11 (3.1)	7 (1.5)	11 (2.3)	0.196	0.651
Length of hospital stay, days [IQR]	7 [4.00, 9.00]	6 [5.00, 9.00]	7 [5.00, 9.00]	0.468	0.739
Admission to the intensive care unit	76 (21.5)	89 (19.3)	101 (21.5)	0.491	>0.999
Length of stay in the intensive care unit, days [IQR]	5 [4.00, 9.00]	5 [3.00, 7.00]	5.5 [4.00, 8.00]	0.344	0.980

p^1: season 2021-2022 vs. season 2018-2019
p^2: season 2022-2023 vs. season 2018-2019
