# Supplementary material for: Severity of respiratory syncytial virus through the COVID-19 pandemic among infants aged ≤2 months: a secondary analysis of the IRIDE cohort study
Source: Pediatr Res. 2025 Jun 10;99(2):663–9. doi: 10.1038/s41390-025-04161-3 (PMC12956566; doi:10.1038/s41390-025-04161-3)
Supplement: Supplementary file 2 — IRIDE_studygroup [file 41390_2025_4161_MOESM2_ESM.pdf]

Severity of Respiratory Syncytial Virus through the COVID-19 pandemic among infants aged ≤2 months: a secondary analysis of the IRIDE cohort study

IRIDE study group members:

Roberta Barachetti,[1] Claudia Pagliotta,[1] Silvia Gulden,[1] Francesco Maria Risso,[2] Michael Colpani,[2] Salvatore Aversa,[2] Paolo Tagliabue,[3] Federico Cattaneo,[3] Roberta Corbetta,[3] Maria Luisa Ventura,[3] Stefano Ghirardello,[4] Ilaria De Lucia,[4] Francesca Garofoli,[4] Luca Mancini,[5] Giulia Angela Carla Pattarino,[5] Costantino De Giacomo,[5] Salvatore Barberi,[6] Mario Vernich,[6] Elisabetta Veronelli,[6] Emanuela Brazzoduro,[6] Ilaria Bottino,[7] Tiziana Varisco,[7] Patrizia Calzi,[7] Alessandro Porta,[8] Paola Alga,[8] Laura Cozzi,[8] Francesca Lizzoli,[8] Lorenzo D'Antiga,[9] Angelo Mazza,[9] Fabiana Di Stasio,[9] Giovanna Mangili,[9] Gian Luigi Marseglia,[10] Amelia Mascolo,[10] Matea Jankovic,[10] Lidia Decembrino,[11] Dario Pantaleo,[11] Chiara Vimercati,[12] Martha Caterina Faraguna,[12] Francesca Cattaneo,[12] Irene Lepri,[12] Laura Pogliani,[13] Liana Bevilacqua,[13] Luca Bernardo,[14] Sergio Arrigoni,[14] Giuseppe Mercurio,[14] Costanza Paramithiotti,[15] Elisabetta Salvatici,[15] Giuseppe Banderali,[15] Alberto Fabio Podestà,[16] Elisa Dusi,[16] Teresa Vivaldo,[16] Sonia Bianchini,[16] Paolo Del Barba,[17] Graziano Barera,[17] Claudia Aracu,[17] Stefano Martinelli,[18] Alice Proto,[18] Marco Fossati,[18] Lorella Rossi,[19] Emilio Palumbo,[19] Marta Odoni,[20] Dalla Verde Ilaria,[20] Ahmad Kantar,[20] Paola Sindico,[21] Grazia Morandi,[21] Valeria Fasolato,[21] Germana Viscogliosi,[22] Nunzia Managanelli,[22] Giuseppe Riva,[22] Chryssoula Tzialla,[23] Roberta Giacchero,[24] Caterina Sabatini,[24] Elena Rossi,[24] Cesare Antonio Ghitti,[25] Ilaria Pacati,[25] Raffaele Badolato,[26] Laura Dotta,[26] Antonella Meini,[26] Ilia Bresesti,[27] Antonio Francone,[27] Anna Maria Plebani,[27] Massimo Agosti,[27] Marco Sala,[28] Simona Santucci,[28] Chiara Cuzzupè,[28] Cristina Bellan,[29] Federica Pontiggia[29], Alice Romero,[30] Chiara Perazzi,[30] Anna Banfi,[30] Gianvincenzo Zuccotti,[31,32] Gianluca Lista[33]

[1]Ospedale Sant'Anna di Como, Como, Italy
[2]Neonatal intensive care unit, Ospedali Civili di Brescia, Brescia, Italy
[3]Fondazione IRCCS San Gerardo dei Tintori di Monza, Italy
[4]S.C. Neonatologia e Terapia Intensiva Neonatale, Fondazione IRCCS Policlinico San Matteo, Pavia, Italy
[5] S.C. Pediatria, ASST Grande Ospedale Metropolitano Niguarda di Milano, Milan, Italy
[6]ASST Rhodense Rho and Garbagnate Milanese, Italy
[7]ASST Brianza - Ospedale Pio XI Desio, Italy
[8]ASST Ovest Milanese, Ospedale Fornaroli di Magenta, Italy
[9]SS Pediatria Internistica, SC Pediatria generale, ASST Papa Giovanni XXIII, Bergamo, Italy
[10]Pediatria, Fondazione IRCCS Policlinico San Matteo e non Ospedale Fondazione IRCCS, Italy
[11]ASST Pavia, Ospedale Civile di Vigevano, Italy
[12]UO Pediatria, Ospedale San Gerardo Di Monza, Italy
[13]ASST OVEST MI Ospedale di Legnano, Italy
[14]ASST FateBeneFratelli Macedonio Melloni - Presidio Ospedaliero Macedonio Melloni di Milano, Italy

[15]SC Pediatria, Presidio San Paolo, ASST Santipaolocarlo, Milan, Italy
[16]ASST Santi Paolo Carlo, Ospedale San Carlo di Milano, Milan, Italy
[17]Pediatric Department, IRCCS San Raffaele Hospital, Milan, Italy
[18]S.C. Neonatologia e Terapia Intensiva Neonatale, ASST Grande Ospedale Metropolitano Niguarda di Milano, Milan, Italy
[19]ASST Valtellina-Alto Lario, Presidio Ospedaliero Di Sondrio, Italy
[20]Gruppo San Donato, Policlinico Ponte San Pietro di San Pietro, Italy
[21]ASST Mantova, A. O. Istituti Ospitalieri Carlo Poma di Mantova, Mantua, Italy
[22]Pediatria, Fondazione Poliambulanza Istituto Ospedaliero di Brescia, Italy
[23]ASST Pavia - Ospedale Civile di Voghera, Italy
[24]ASST Lodi, Ospedale Maggiore di Lodi, Italy
[25]ASST Bergamo Est - Ospedale "Bolognini" di Seriate, Italy
[26]ASST Degli Spedali Civili di Brescia, Brescia, Italy
[27]ASST Settelaghi, Ospedale “F. Del Ponte”, University of Insubria, Varese, Italy
[28]ASST della Brianza, Ospedale di Vimercate, Italy
[29]Terapia Intensiva Neonatale - ASST Bergamo Est Ospedale Bolognini Seriate Italy
[30]Pediatric Department, "Vittore Buzzi" Children's Hospital, Università degli Studi di Milano, Milan, Italy
[31]Pediatric Department, "Vittore Buzzi" Children's Hospital, Milan, Italy
[32]Department of Biomedical and Clinical Sciences, Università degli Studi di Milano, Milan, Italy
[33]Division of Neonatology, "Vittore Buzzi" Children's Hospital, Milan, Italy
